# Effect of taurine chloramine on the production of matrix metalloproteinases (MMPs) in adiponectin- or IL-1β-stimulated fibroblast-like synoviocytes

**DOI:** 10.1186/1423-0127-17-S1-S27

**Published:** 2010-08-24

**Authors:** Kyoung Soo Kim, Hyun-Mi Choi, Da Hee Oh, Chaekyun Kim, Jin Seok Jeong, Myung Chul Yoo, Hyung-In Yang

**Affiliations:** 1 East-West Bone & Joint Research Center, East-West Neo Medical Center, Kyung Hee University, 149 Sangil-dong, Gangdong-gu, Seoul, 137-727, Republic of Korea; 2 Center for Advanced Medical Education by BK21 Project, Inha University School of Medicine, Incheon, Republic of Korea; 3 Divison of Rheumatology, Department of Internal Medicine, East-West Neo Medical Center, Kyung Hee University, 149 Sangil-dong, Gangdong-gu, Seoul, 137-727, Republic of Korea; 4 Department of Orthopedic Surgery, East-West Neo Medical Center, Kyung Hee University, 149 Sangil-dong, Gangdong-gu, Seoul, 137-727, Republic of Korea; 5 Research Institute, Dong-A Pharm. Co., Ltd., 47-5 Sangal-dong, Yongin-si, Kyunggi-do, 446-905, Republic of Korea

## Abstract

**Background:**

Adiponectin greatly stimulated the expression of matrix metalloproteinases (MMPs) in fibroblast-like synoviocytes (FLSs) as did IL-1β. We wondered whether taurine chloramine (TauCl) inhibits the production of MMPs stimulated by adiponectin in the same pattern as by IL-1β stimulation *in vitro*

**Methods:**

Synovial cells from rheumatoid arthritis (RA) patients were treated with adiponectin or interleukin (IL)-1β for 24 hr in the presence or absence of TauCl. The culture supernatant was collected and the levels of MMPs were measured by enzyme-linked immunosorbent assay (ELISA). The IκB signaling pathways stimulated by adiponectin were studied and the levels of NF-κB in the nuclei of the cells were analyzed by ELISA.

**Results:**

TauCl (600 µM) inhibited MMP-13, but not MMP-1, expression in IL-1β-stimulated RA FLSs. However, TauCl at the same concentration significantly inhibited the production of both adiponectin-stimulated MMP-1 and MMP-13 expression. TauCl inhibited the degradation of IκB-α stimulated by adiponectin, but not by IL-1β. Similarly, the level of NF-κB in the nucleus was increased by adiponectin stimulation and was inhibited by 600 µM TauCl. However, the levels of NF-κB increased by IL-1β stimulation were not inhibited by 600 µM TauCl.

**Conclusions:**

TauCl more effectively inhibited MMPs expression induced by adiponectin than that by IL-1β in RA FLS, suggesting that TauCl plays an important role in down-regulating the expression of MMPs in arthritic joints.

## Background

One of the characteristics of rheumatoid arthritis (RA) is the infiltration of inflammatory immune cell types into the synovial fluid of the joints. Proliferative fibroblast-like synoviocytes (FLS) play crucial roles in the propagation of inflammation because they produce many mediators of inflammation [1]. Immune cells recruited into joint cavities by FLS also contribute to the progressive destruction of the cartilage in distal joints [2]. Among the detrimental immune cells present in RA joints, neutrophils have been a primary focus of research in RA due to their number and function [3-5]. Polymorphonuclear neutrophils (PMNs) are usually thought of as the leukocyte population involved in the acute inflammatory response, acting as a first line of defense against invading microorganisms [6]. As such, neutrophils are crucial for pathogenesis defense as part of the innate immunity. However, neutrophils can themselves produce an array of inflammatory mediators, including cytokines, chemokines, and complement [7]. In addition, they release granule-packaged proteases such as matrix metalloproteinases (MMPs) and reactive oxygen intermediates such as nitric oxide (NO) and hypochlorous acid (HOCl) for intracellular digestion during phagocytosis [8,9]. Thus, neutrophils seem to play an important role in the pathogenesis of RA [5].

In particular, HOCl, which is produced by the myeloperoxidase-H_2_O_2_-Cl system in the phagocytotic process of neutrophils, reacts with amino acids, carbohydrates, nucleic acids and lipids and thus contributes to the mutagenic and cytotoxic effects of phagocytes on microbial pathogens [10]. After clearance of pathogens, the accumulated HOCl should be removed because a high concentration of HOCl can affect the host tissue. Thus, activated neutrophils have developed homeostatic mechanisms for neutralizing the cytotoxic HOCl by producing high levels of taurine, which is one of the most abundant free intracellular amino acids in mammalian tissue and blood cells. Taurine reacts with HOCl to form taurine chloramine (TauCl) by acting as a scavenger of HOCl [11]. Most notably, TauCl has been shown to play a major role in down-regulating the expression of inflammatory mediators such as chemokines, cytokines, cyclooxygenase-2 (COX-2), and inducible nitric oxide synthase (iNOS) in different cell types [12-15]. TauCl also differentially inhibits the expression of MMP-1 and MMP-13, which play dominant roles in RA and osteoarthritis (OA), in IL-1β-stimulated fibroblast-like synoviocytes (FLS) [16].

Adipose tissue, once viewed as simply a storage and release depot for lipids, is now considered an endocrine tissue [17,18] that secretes various substances (adipokines) including tumor necrosis factor-α (TNF-α), interleukin-6 (IL-6), leptin, adiponectin, resistin, visfatin, omenetin, and others [19,20]. Recent findings suggest that adiponectin may be involved in the pathogenesis of rheumatoid arthritis (RA). However, the role of adiponectin in the pathogenesis of RA is still controversial because of conflicting reports about its function. Recently we found that adiponectin may contribute to synovitis and joint destruction in patients with RA by stimulating VEGF, MMP-1, and MMP-13 expression more than proinflammatory mediators in fibroblast-like synoviocytes [21]. However, it remains to be determined whether adiponectin or IL-1β contributes more to the increased expression of these MMPs in arthritic joints and it also should be determined whether TauCl inhibits the production of MMPs stimulated by adiponectin and those stimulated by IL-1β *in vitro*. In this study, we investigated the specific inhibitory effect of TauCl by showing that TauCl differentially inhibits MMPs expression in IL-1β- or adiponectin-stimulated FLSs.

## Methods

### Primary fibroblast-like synoviocytes (FLS) culture

After obtaining informed consent, synovial tissues were collected from RA patients who met the 1987 American College of Rheumatology criteria for the diagnosis of RA and who were undergoing therapeutic joint surgery. FLS were isolated as described previously [16]: tissues were digested with gentle shaking in 20 ml of RPMI 1640 (Gibco-BRL, Grand Island, NY, USA) containing 1 mg/ml collagenase (Gibco-BRL) at 37°C for 90 min, filtered through a 70-μm cell strainer, and cultured in 75-cm^2^ culture flasks with Dulbecco’s Modified Essential Medium (DMEM; Gibco-BRL) supplemented with 20% (v/v) fetal bovine serum (FBS; Gibco-BRL) and 1× antibiotic-antimycotic (Gibco-BRL). After the cells had grown to confluence, they were detached with 0.25% trypsin (Gibco-BRL) and split at a 1:4 ratio. FLS were used at passages 3-6 for all experiments. Visual examination of cell morphology under light microscopy and FACS analysis of cells stained with anti-CD11b antibody (Santa Cruz Biotechnology, Santa Cruz, CA, USA) confirmed that FLS accounted for more than 95% of the cells.

### Preparation of TauCl

TauCl was synthesized by mixing equimolar amounts of NaOCl (Aldrich Chemical, Milwaukee, MI, USA) and taurine (Sigma, St. Louis, MO, USA). TauCl formation was verified by ultraviolet (UV) absorption (200–400 nM) [22]. Endotoxin-free or low-endotoxin grade water and buffers were used. Stock solutions of TauCl were kept at 4°C and used within 3 days.

### Enzyme-linked immunosorbent assay (ELISA)

The levels of MMP-1 and MMP-13 secreted in the culture media by IL-1β-stimulated FLS (2.5 × 10^5^ cells/60-mm dish/2-ml serum-free media) in the presence or absence of TauCl were measured by enzyme-linked immunosorbent assay (ELISA; R&D Systems, Inc., Minneapolis, MN, USA). For the measurement of transcription factors, NF-κB and AP-1, in the nucleus, FLSs were seeded (5 × 10^6^ cells) into 100-mm dishes and grown to 80% confluence. The cells were serum-starved overnight and stimulated by adiponectin (10 µg/ml) or IL-1β (10 ng/ml) for 120 min and 90 min, respectively, in the presence or absence of TauCl (600 µM). The cells were then washed twice in PBS and treated with lysis buffer; the transcription factors were extracted from the nucleus according to the manufacturer’s protocol (Active Motif, Seoul, Korea).

### Semi-quantitative RT-PCR

After the culture supernatants were harvested, the cells were used for semi-quantitative RT-PCR. TRIzol® reagent (Invitrogen) was used to extract total RNA from the cells. Complementary DNA was synthesized from 1 μg of total RNA in 20 μl of reverse transcription reaction mixture containing 5 mM MgCl_2_, 1× RT buffer, 1 mM dNTP, 1 U/µl of RNase inhibitor, 0.25 U/μl of AMV reverse transcriptase, and 2.5 μM random 9-mers. For semi-quantitative PCR, aliquots of cDNA were amplified with the primers in a 25-μl PCR mixture containing 1× PCR buffer, 0.625 units of *TaKaRa* Ex Taq^TM^ HS, and 0.2 μM of specific upstream primers, according to the manufacturer’s protocol (TaKaRa Bio, Kyoto, Japan). The PCR conditions for the MMPs were as follows: 30–33 cycles of 95° C for 45 sec, 55–60° C for 45 sec, and 72° C for 45 sec. PCR products were subjected to electrophoresis in 1.5% agarose gels containing ethidium bromide, and the bands were visualized under UV light. The primers were synthesized by Bioneer Co. Ltd. (Seoul, Republic of Korea); their sequences are listed in Table 1.

**Table 1:** Sequences of PCR primers used in this study

Primer name	Primer sequence	Product size
MMP-1 sense	5′-CCT AGC TAC ACC TTC AGT GG-3′	
MMP-1 antisense	5′-GCC CAG TAC TTA TTC CCT TT-3′	338 bp

MMP-13 sense	5′-TTG AGG ATA CAG GCA AGA CT-3′	
MMP-13 antisense	5′-TGG AAG TAT TAC CCC AAA TG-3′	311 bp

β-actin sense	5′-TCA TGA GGT AGT CAG TCA GG-3′	
β-actin antisense	5′-CTT CTA CAA TGA GCT GCG TG-3′	305 bp

### Western blot analysis

FLS cultured (5 × 10^5^ cells) in 60-mm dishes were serum-starved overnight and stimulated by IL-1β (10 ng/ml) or adiponectin (10 µg/ml) for 30 min or 60 min, respectively, in the presence or absence of TauCl. The cells were subsequently washed twice in PBS and treated with 50 μl of lysis buffer [20 mM Tris–Cl (pH 8.0), 150 mM NaCl, 1 mM EDTA, 1% Triton X-100, 20 µg/ml chymostatin, 2 mM phenylmethylsufonyl fluoride (PMSF), 10 µM leupeptin, and 1 mM 4-(2-aminoethyl)benzenesulfonyl fluoride (AEBSF)]. Cells were scraped using a rubber policeman prior to the addition of another 50 μl of lysis buffer. The cells were transferred to a microcentrifuge tube, incubated on ice for 30 min with occasional agitation every 5 min, and centrifuged for 15 min at 12,000 rpm (16,090 × g), and the supernatant was then analyzed for protein concentration using the Bio-Rad Protein Assay Kit (Bio-Rad, Hercules, CA, USA). Thirty µg of cytoplasmic protein extract were then boiled in 5× Laemmli sample buffer for 5 minutes. The samples were separated by 12% SDS-PAGE and transferred to Hybond-ECL membranes (Amersham, Arlington Heights, IL, USA). The membranes were blocked with 6% nonfat milk dissolved in TBST buffer [10 mM Tris–Cl (pH 8.0), 150 mM NaCl, 0.05% Tween 20]. The blots were probed with rabbit polyclonal antibodies against IκBα and β-actin (Cell Signaling Technology, Beverly, MA, USA) diluted 1:1000 in TBS for 2 h and then incubated with 1:1000 dilution of horseradish peroxidase-conjugated goat anti-rabbit IgG secondary antibody. The blots were developed using the ECL method (Amersham). For re-probing, the blots were incubated in stripping buffer [100 mM 2-mercaptoethanol, 2% SDS, 62.5 mM Tris–HCl (pH 6.7)] at 50° C for 30 min with occasional agitation.

### Statistical analysis

All experiments were repeated three times, and the results are expressed as the mean ± standard deviation. Statistical evaluation was performed by the Mann-Whitney test. Differences were considered statistically significant at *P*<0.05.

## Results

### Differential inhibition of the production of pro-MMP1- and pro-MMP-13 by taurine chloramine in adiponectin- or IL-1β-stimulated FLSs

**Figure 1:**
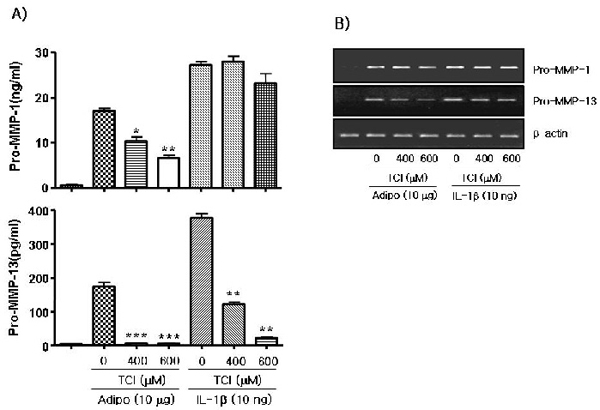
**Effect of taurine chloramine on the expression of MMPs in adiponectin- or IL-β-stimulated FLSs.** The expressions of the collagenases (MMP-1 and MMP-13) were determined by ELISA [A], and semi-quantitative RNA analysis [B]. Synovial cells (2.5 × 10^5^ cells/60-mm dish/2-ml serum-free media) were treated with taurine chloramine (TauCl) for 30 min prior to 24 h of adiponectin (10 µg/ml) or IL-1β (10 ng/ml) stimulation for analysis of MMP proteins by ELISA. Supernatants were collected for ELISA and the cells were used for RNA extraction for semi-quantitative RT-PCR. Experiments were performed in quadruplicate with cells from patients. The experiments were repeated with FLSs from three patients. The data shown are representative of 3 independent experiments, and similar results were obtained from all 3. Values are expressed as mean ± S.E.M. *P<0.05, **P<0.01, and *** P<0.001 versus non-treated group.

To investigate whether TauCl inhibits the production of MMPs stimulated by adiponectin in the same pattern as it inhibits that by IL-1β *in vitro*, fibroblast-like synoviocytes (FLSs) were cultured and stimulated with adiponectin (10 µg/ml) or IL-1β (10 ng/ml) in the presence or absence of 600 µM TauCl (Fig. 1A). Adiponectin and IL-1β significantly stimulated the expression of pro-MMP-1 and pro-MMP-13 in FLSs. Similarly, the mRNA level of pro-MMPs was also significantly increased in the FLSs. TauCl (600 µM) could not inhibit the IL-1β-induced increase in pro-MMP-1; however, it significantly inhibited the level of pro-MMP-13 by about 3-fold, even at 400 µM. At 600 µM, TauCl also inhibited the level of pro-MMP-13 by about 10-fold in IL-1β-stimulated FLSs as shown previously [16]. In contrast, TauCl at 400 µM significantly inhibited the level of pro-MMP-1 in adiponectin-stimulated FLSs and further inhibited the expression of pro-MMP-1 by about 2-fold at 600 µM. In addition, even 400 µM TauCl greatly inhibited the expression of pro-MMP-13 to the basal expression level of non-stimulated FLSs. Similarly, the mRNA level of pro-MMPs was inhibited by TauCl as shown in Fig. 1B.

### Effect of taurine chloramine on the degradation of IκB-α by adiponectin or IL-1β stimulation

**Figure 2:**
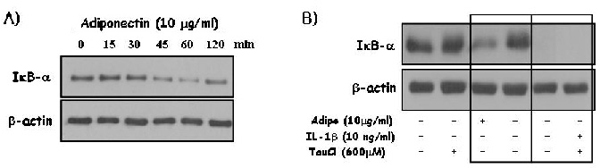
**Effect of taurine chloramine on the signaling pathways stimulated by adiponectin.** (A) Synovial cells (5 × 10^5^ cells/60-mm dish/2-ml serum-free media) were treated with adiponectin (10 µg/ml). Time courses of the signaling pathways activated during adiponectin stimulation. (B) Synovial cells (5 × 10^5^ cells/60-mm dish/2-ml serum-free media) were treated with TauCl 30 min prior to 30- or 45-min adiponectin (10 µg/ml) or IL-1β (10 ng/ml) stimulation, respectively. TauCl (600 µM) significantly inhibited the IκB-NF-κB signaling pathway by inhibiting the degradation of IκB-α induced by adiponectin, but not that by IL-1β. The data shown are representative of 3 independent experiments, and similar results were obtained from all 3.

To explain the differential effect of TauCl on the production of pro-MMPs in FLSs, the signaling pathway was investigated in adiponectin-stimulated FLSs. First, the signaling pathway was investigated in a time course and compared with that of IL-1β (Fig. 2). Adiponectin seems to activate the IκB kinase signaling pathway, but it did not affect the mitogen-activated protein kinase (MAPK) (Fig. 2A). The degradation of IκB-α was the highest at 60 min of adiponectin stimulation. The time course change of IκB-α degradation during adiponectin stimulation was different from that of IL-1β as reported previously elsewhere [16]. More importantly, the IκB-α degradation induced by adiponectin was greatly inhibited by 600 µM TauCl. However, the degradation of IκB-α stimulated by IL-1β was not affected by 600 µM TauCl.

### Effects of taurine chloramine on NF-κB migration into the nucleus by adiponectin or IL-1β stimulation

**Figure 3:**
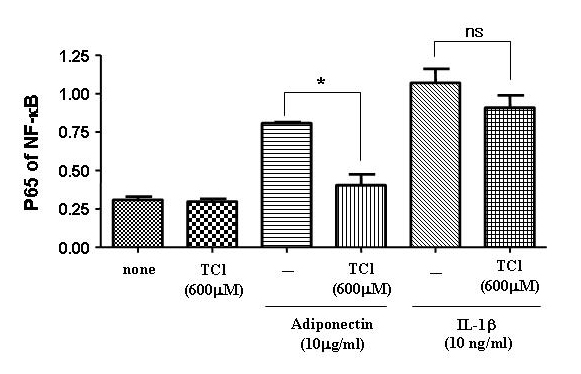
**Effects of taurine chloramine on transnuclear migration of NF-κB.** FLSs were seeded (5 × 10^6^ cells) into 100-mm dishes and grown to 80% confluence. The cells were serum-starved overnight and stimulated by adiponectin (10 µg/ml) or IL-1β (10 ng/ml) for 120 min and 90 min, respectively, in the presence or absence of TauCl (600 µM). The nuclear levels of NF-κB were measured by ELISA detection of p65 in nuclear extracts. TauCl differentially inhibited the level of NF-κB in the nuclei of adiponectin- or IL-1β-stimulated RA FLSs. Values are expressed ± S.E.M. *p<0.05 versus adiponectin- or IL-1β-treated group in the presence of TauCl. ns, not significant.

To assess whether the inhibition of IκB signaling pathways by TauCl also inhibits the migration of NF-κB into the nucleus, the levels of the transcription factors NF-κB were measured in adiponectin- or IL-1β-stimulated FLSs in the presence or absence of TauCl. As shown in Fig. 3, the level of NF-κB was increased in the nuclei of adiponectin- or IL-1β-stimulated FLSs. At 600 µM, TauCl significantly inhibited the migration of NF-κB into the nuclei of adiponectin-stimulated FLSs. However, it slightly inhibited the migration of NF-κB into the nuclei of IL-1β-stimulated FLSs with no statistical significance, even though it did not inhibit the degradation of IκB-α. All of these results suggest that TauCl plays a more important role in down-regulating the expression of MMPs in arthritic joints than expected.

## Discussion

MMPs, which are primarily produced in fibroblast-like synoviocytes (FLS) in RA, are proteases that participate in irreparable proteolytic degradation and the remodeling of the extracellular matrix. According to their substrate specificities, primary structures, and cellular localizations, MMPs can be classified into five main groups: collagenases (MMP-1, MMP-8, MMP-13), gelatinases (MMP-2, MMP-9), stromelysins (MMP-3, MMP-10), matrilysins (MMP-7, MMP-26), and membrane-bound MT-MMPs (MMP-14, MMP-15, MMP-16, MMP-17, MMP-24, MMP-25) [23]. In particular, the MMP-1 and MMP-13 collagenases play dominant roles in RA and osteoarthritis. The collagenases have the unique ability to cleave the triple helix of collagen, thereby allowing the chains to unwind; the chains then become susceptible to further degradation by other MMPs such as gelatinase (MMP-2 and MMP-9). Thus, the collagenases can be regarded as rate-limiting components of the collagen degradation process [24,25]. In addition, MMP-13 is a potent protease that is capable of degrading a wide range of collagenous and noncollagenous extracellular matrix macromolecules [26,27]. MMP-13 is remarkably active against collagen type II, the predominant collagen in cartilage [28]. Thus, it is necessary to understand the factors to control the expression of these collagenases in arthritic joints.

It has not yet been determined whether adiponectin or IL-1β contributes more to the increased expression of these MMPs in arthritic joints, but the high concentration of adiponectin in joint fluids of patients with arthritis seems to contribute significantly to the joint degradation. In this study, TauCl showed a greater inhibitory effect on the expression of MMP-1 and MMP-13 induced by adiponectin than by IL-1β. Thus, TauCl may down-regulate the expression of MMPs in arthritic joints, considering that the physiological concentration of adiponectin is high in arthritic joints. The inhibition of MMP-13 expression with lower concentrations of TauCl would also be a potentially effective strategy for controlling the destruction of joint cartilage in RA and OA. In addition, TauCl may be produced as a part of the homeostatic response to infection and inflammation, thus playing a critical role in limiting the duration and intensity of immune inflammation [15]. In support of this hypothesis, synovial fluid neutrophils of RA patients showed impaired generation of TauCl [29]; thus, the involvement of TauCl in the pathogenesis of arthritis should be studied further.

To elucidate the molecular mechanisms by which TauCl more effectively inhibits adiponectin-induced MMPs expression than that induced by IL-1β, the levels of MAPK phosphorylation and IκB degradation in adiponectin-stimulated FLSs were compared with those of IL-1β. Adiponectin did not stimulate the phosphorylation of ERK1/2, p38, or JNK in the MAP kinase signaling pathways, but rather stimulated the signaling pathways of IκB kinase through IκB-α degradation. In contrast, IL-1β stimulated both pathways as reported previously [16]. At 600 µM, TauCl did not significantly inhibit IκB degradation stimulated by IL-1β, but greatly inhibited the degradation stimulated by adiponectin. Meanwhile, we previously reported that partial inhibition of IκB-α degradation was seen with 600 µM TauCl, as evidenced by NF-κB immunostaining in both the cytoplasm and the nucleus, and IκB-α degradation was completely inhibited by 800 µM TauCl, thereby preventing the migration of NF-κB into the nucleus [16]. In this study, however, the inhibition of adiponectin-mediated IκB-α degradation by TauCl did not significantly inhibit the migration of NF-κB into nucleus. In addition, at 600 µM, TauCl did not show an inhibitory effect on IκB-α degradation in IL-1β stimulation based on Western blot analysis, but the IL-1β-induced migration of NF-κB into nucleus was partially inhibited. This may indicate that TauCl partially inhibits the activation of NF-κB in IL-1β stimulation through unknown mechanisms. In other words, signaling pathways other than the IκB kinase pathway may be affected by adiponectin or IL-1β and be involved in the stimulation of MMP-1 and MMP-13 through other transcription factors in addition to NF-κB. For example, protein kinase C (PKC) delta is known to play a key role in the stimulation of MMP-13 via cross-talk with MAP kinases in basic fibroblast growth factor (bFGF)-stimulated human adult articular chondrocytes [30]. The differential effects of TauCl on the expression of MMPs induced by adiponectin or IL-1β may also be related to other transcription factors that are differentially activated by adiponectin versus IL-1β. Many transcriptional binding sites, such as AP-1, Ets/polyomavirus enhancer 3 (OS-2) and Runx2 sites, have been identified in the human MMP-13 proximal promoter [31-33]. An AG-rich element (AGRE) regulatory site was recently found in the human MMP-13 proximal promoter [34]. These or other transcription factors may therefore differentially contribute to the increased expression of MMPs in adiponectin- or IL-1β-stimulated FLS. TauCl may differentially inhibit the activation of these transcription factors by adiponectin or IL-1β.

TauCl is less toxic than its precursor HOCl/OCl^-^, but cytotoxic effects of TauCl at high concentrations have been reported. Its toxicity seems to differ between cell types [35]. Kontny *et al*. reported that TauCl caused progressive necrosis of RA FLS at ≥500 µM [36]. However, in our previous report, TauCl toxicity seemed to vary according to the individual RA patient [16]. In addition, different cell passages may contribute to the variance in sensitivity to TauCl, since RA FLS show different characteristics according to passage [37,38]. Thus, the differential inhibitory effect of TauCl may indirectly indicate that TauCl inhibits a specific reaction in adiponectin- or IL-1β-stimulated FLSs rather than a cytotoxic or nonspecific reaction.

## Conclusions

TauCl more effectively inhibited MMPs expression induced by adiponectin than that by IL-1β in RA FLS, suggesting that TauCl plays an important role in down-regulating the expression of MMPs in arthritic joints. Furthermore, its mode of action is based on a specific reaction, rather than a cytotoxic or nonspecific reaction.

## Competing interests

The authors declare that they have no competing interests.

## Authors' contributions

Kim KS and Yang HI participated in the data analysis and the design of the study, and drafted the manuscript. Choi HM, Oh DH, Jeong JS and Kim C performed the experiments. Yoo MC provided the synovium from patients and participated in the design of the study. All authors read and approved the final manuscript.
